# A review on cell-free RNA profiling: Insights into metabolic diseases and predictive value for bariatric surgery outcomes

**DOI:** 10.1016/j.molmet.2024.101987

**Published:** 2024-07-06

**Authors:** Manvita Mareboina, Elen Deng, Ioannis Mouratidis, Nelson S. Yee, Nelly Pitteloud, Ilias Georgakopoulos-Soares, Dionysios V. Chartoumpekis

**Affiliations:** 1 Institute for Personalized Medicine, Department of Biochemistry and Molecular Biology, The Pennsylvania State University College of Medicine, Hershey, PA, USA; 2 Division of Hematology-Oncology, Department of Medicine, Penn State Health Milton S. Hershey Medical Center, Next-Generation Therapies Program, Penn State Cancer Institute, Hershey, PA, USA; 3 Service of Endocrinology, Diabetology and Metabolism, Lausanne University Hospital and University of Lausanne, CH-1011, Lausanne, Switzerland

**Keywords:** cfRNA, Metabolic outcomes, Obesity, Comorbidities, Metabolic surgery, Diabetes, Dyslipidemia, Fatty liver disease

## Abstract

**Background:**

The advent of liquid biopsies presents a novel, minimally invasive methodology for the detection of disease biomarkers, offering a significant advantage over traditional biopsy techniques. Particularly, the analysis of cell-free RNA (cfRNA) has garnered interest due to its dynamic expression profiles and the capability to study various RNA species, including messenger RNA (mRNA) and long non-coding RNA (lncRNA). These attributes position cfRNA as a versatile biomarker with broad potential applications in clinical research and diagnostics.

**Scope of Review:**

This review delves into the utility of cfRNA biomarkers as prognostic tools for obesity-related comorbidities, such as diabetes, dyslipidemia, and non-alcoholic fatty liver disease.

**Major conclusions:**

We evaluate the efficacy of cfRNA in forecasting metabolic outcomes associated with obesity and in identifying patients likely to experience favorable clinical outcomes following bariatric surgery. Additionally, this review synthesizes evidence from studies examining circulating cfRNA across different physiological and pathological states, with a focus on its role in diabetes, including disease progression monitoring and treatment efficacy assessment. Through this exploration, we underscore the emerging relevance of cfRNA signatures in the context of obesity and its comorbidities, setting the stage for future investigative efforts in this rapidly advancing domain.

## 1 Background

In recent years, there has been a surge in interest in minimally-invasive methods for biomarker discovery for the early detection and monitoring of different human diseases. Liquid biopsies are a promising emerging alternative to traditional invasive diagnostic methods, such as excisional biopsy. A liquid biopsy refers to a medical test which analyzes fluid non-invasively, with urine, tears, sweat, and semen, or invasively, with blood, cerebrospinal fluid, or pleural fluid [1,2] to obtain information about a person's health or disease status by examining profiles of biomolecules including DNA, RNA, proteins, exosomes or circulating tumor cells. Liquid biopsies offer significant advantages due to their minimally invasive nature, enabling the simultaneous detection of multiple markers indicative of various pathologies. This approach is particularly valuable in scenarios where tissue samples are scarce, difficult to procure, or when continuous monitoring is necessitated [3]. An added benefit of liquid biopsies is that they further omit the need for solid tissue sampling and may be used for genotyping if tissue testing is inadequate [4].

**Figure 1:**
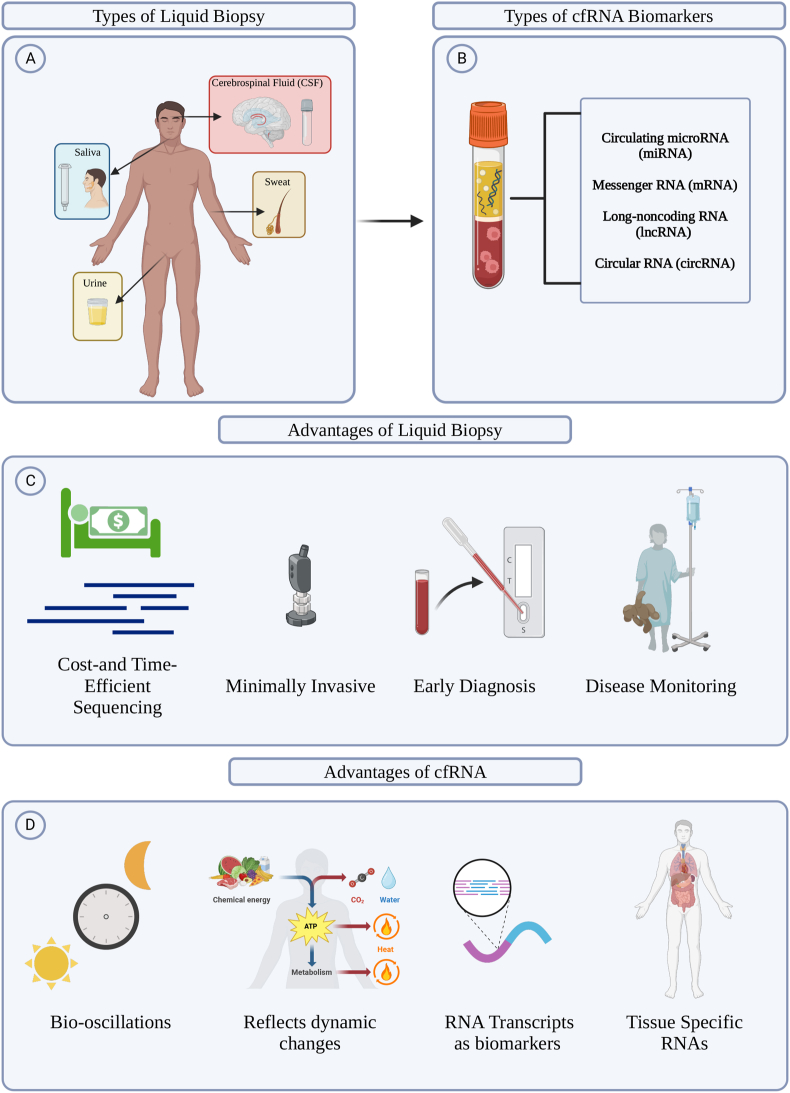
**(A)** Different types of liquid biopsy that can be utilized are displayed on the human figure. **(B)** The various cfRNA biomarkers that can be measured from liquid biopsies are shown. **(C)** Figure depicts the benefits of liquid biopsy. **(D)** Advantages of cfRNA utilization in liquid biopsy. Created with BioRender.com.

In the context of cfRNA, a liquid biopsy involves the collection and examination of RNA molecules found in bodily fluids outside cells, such as in blood, providing insights into various physiological and pathological conditions without the need for traditional tissue biopsies [1,5]. cfRNA can be passively released from cells due to tissue injury, chronic inflammation, apoptosis, or necrosis, as well as from cells with short half-lives like platelets. cfRNA can be actively secreted, either via exosomes and microparticles, or conjugated with lipoproteins or RNA-binding proteins [6]. The first consideration of using cfRNA as a biomarker was conducted by Larson et al. which was the first transcriptome-wide characterization of cfRNA in cancer and non-cancer individuals. Results revealed ‘dark channel biomarker’ genes that are recurrently detected in cancer patients, indicating that cfRNA has the potential to detect cancer, cancer subtype, and predict its origin [7]. Other studies have also found that cfRNA profiles can elucidate other human diseases such as Alzheimer's, Parkinson's, embryonic congenital defects [[8], [9], [10], [11]]. The distinctive attributes of cfRNA present noninvasive diagnostic opportunities, showcasing considerable potential for diagnosing various pathological conditions, including applications in prenatal screening and disease diagnostics (Figure 1) [12]. cfRNA-based biomarkers have the potential to offer a cost-effective clinical tool, due to the ease of sample collection and the rapidly decreasing sequencing costs [5].

This review centers on the exploration of circulating blood biomarkers in metabolic diseases, with a specific focus on the dynamic RNA expression landscape. Notably, we delve into the multifaceted role of cfRNA as a diagnostic tool across various clinical scenarios, encompassing early disease detection in states of chronic inflammation and metabolic disease such as fatty liver disease, dyslipidemia, atherosclerosis, obesity, and outcomes post bariatric surgery [12,13].

### 1.1 cfRNA species as biomarkers

The process of cfRNA biomarker discovery involves isolating cfRNA from blood, typically using blood serum or plasma due to their acellular nature, which helps reduce RNA contamination from blood cells [5]. The choice between serum and plasma depends on the specific goals of the study. Initially, blood samples are obtained from individuals, and the cfRNA is isolated and purified. Subsequently, next-generation sequencing (NGS) techniques are employed to analyze the extracted cfRNA, enabling the identification of various RNA molecules. Notably, various small non-coding RNA molecules, including circulating miRNA, messenger RNA (mRNA), long non-coding RNA (lncRNA), circular RNA (circRNA), Piwi-interacting RNA (piRNA), and transfer RNA (tRNA), exhibit distinct expression and release patterns specific to different cell types [[12], [13], [14]]. The cellular origin of cfRNA varies depending on the specific species of RNA. For example, miRNAs are transcribed in the nucleus and exported to the cytoplasm for further catalytic processing. This small molecule alone can regulate gene expression by binding to mRNAs leading to mRNA cleavage, degradation, or repression of translation [15]. Through computational analyses and machine learning, researchers are discerning specific cfRNA signatures associated with different health conditions [5] and are developing alternate approaches to blood-based liquid biopsies, such as urinary biopsies [1]. miRNAs, a class of short non-coding RNA molecules, post-transcriptionally regulate gene expression in eukaryotes, targeting more than 60% of all human genes and playing crucial roles in various physiological and pathological processes [16]. As potential biomarkers, miRNAs are considerably stable, attributed to protection by lipid or protein carriers leading to resistance to RNase degradation, facilitating their detection in biological fluids [6]. During short-term storage and transportation of blood samples, cells can undergo changes like apoptosis and stress response, impacting the expression of the original transcriptome in plasma which contains RNA from various tissues [17]. The separation of plasma from other blood components is usually done with several rounds of centrifugation but plasma samples can still be contaminated with leukocyte, red blood cells and platelet RNA [5,17]. Plasma RNases can also degrade cfRNA, however, previous studies suggest that plasma cfRNA can avoid degradation by being encapsulated in vesicles or forming complexes with proteins or lipids. Plasma cfRNA stored at 4 °C and processed within 6 h was shown to maintain most of the original cfRNA transcriptome [17]. The use of special blood collection tubes with a preservative that stabilizes nucleated blood cells is also gaining popularity in studies with cfRNA as they offer the advantage for keeping the samples for longer times at room temperature facilitating their shipment to the lab for processing [18]. There is also appeal in studying long RNAs (>200 nt), including mRNAs and lncRNAs, reflecting the growing interest in using cfRNA as biomarkers. Despite encountering technical hurdles, such as limited reproducibility stemming from the lack of standardized protocols, the expansive repertoire of known long RNAs intimates a considerable potential for the identification of reliable disease biomarkers [5]. Herein we delve into circulating cfRNA signatures, specifically its applications in individuals with obesity, focusing on their potential as prognostic indicators for associated metabolic comorbidities, including diabetes, dyslipidemia, and fatty liver disease and also as predictors of response to bariatric surgery. cfRNA signatures may serve not only as predictive markers but they could also point to potential molecular intricacies that underlie these clinical conditions. This comprehensive examination of the current landscape of cfRNA signatures in this rapidly evolving field in both human and mouse models aims to consolidate existing knowledge and outline the prospects for future research.

### 1.2 Dynamic physiological cfRNA signature profiles

cfRNA holds clinical potential as a health status indicator from various tissues. However, gaps in understanding the physiologic origins and normal cfRNA signature profiles, including contributing tissues and cell types, still persist [19]. A recent study by Vorperian et al. utilized exome-enriched cell-free transcriptome data to characterize cell-type-specific signals in healthy donor plasma, revealing significant contributions from platelets (26.3%), erythrocyte/erythroid progenitors (24.2%), and leukocytes (immune cells) (12.8%) to cfRNA signature profiles [19]. Bood, brain, liver, and gastrointestinal tract markers were also detectable. The authors also demonstrated the non-invasive detection of cell-type-specific changes in various health conditions, including chronic kidney disease, non-alcoholic fatty liver disease, and Alzheimer's disease, through the measurement of signature scores across cell types [19].

Further, it is important to understand cfRNA's role in regular physiology, particularly its dynamics influenced by circadian rhythms and food intake. In the research conducted by Heegaard et al. an examination of plasma samples from 24 healthy male volunteers explored the circadian rhythmicity of circulating miRNAs. The study identified that approximately one-third of measurable plasma miRNAs demonstrated rhythmic behavior, displaying two primary phase patterns. These findings underscore the importance of accounting for bio-oscillations in miRNA biomarker investigations, highlighting the potential for further investigation into specific circulating miRNAs and their roles in circadian rhythm regulation [20]. The diurnal variation of cfDNA and cfRNA in plasma from healthy volunteers over two days has also been investigated [21]. Results indicated that diurnal cycles and meal consumption have minimal effects on abundance of total cfDNA, total cfRNA, and the selected cfRNA transcripts ACTB and GAPDH that are often used as controls in several studies. Noteworthy individual variations were observed for the GAPDH cfRNA transcript, emphasizing the importance of considering patient-specific baselines in clinical studies [21].

Furthermore, researchers examined the stability of the expression of selected cfRNAs (miRNAs) in human breast milk over the second month of lactation and a 24-hour period with the purpose of identifying good reference genes for studies of cfRNA expression in milk by qPCR [22]. Stable expression of miR-21 and miR-16 was observed in whole milk during the second month of lactation. miR-146b and let-7d were identified as better reference genes in lipid and skim milk fractions, and a daily oscillation of miR-16-5p was found.

Maternal cfDNA and cfRNA have also been commonly used to screen for genetic abnormalities during pregnancy and are thought to have potential in detecting adverse pregnancy outcomes based on placental function. Analysis of cfDNA and cfRNA in maternal and cord plasma samples in maternal obesity have shown specific nucleic acid changes, particularly in the first trimester, that preceded the development of gestational diabetes [23]. Overall, unraveling the complexities of cfRNA in diverse physiological contexts, from its cell-type-specific signals to circadian rhythmicity and responses to dietary influences, contributes valuable insights not only into its potential as a non-invasive health status indicator but also into taking into account these physiological changes when analyzing cfRNA profiles in the context of diseases.

## 2 cfRNA profiles in metabolic diseases

**Figure 2:**
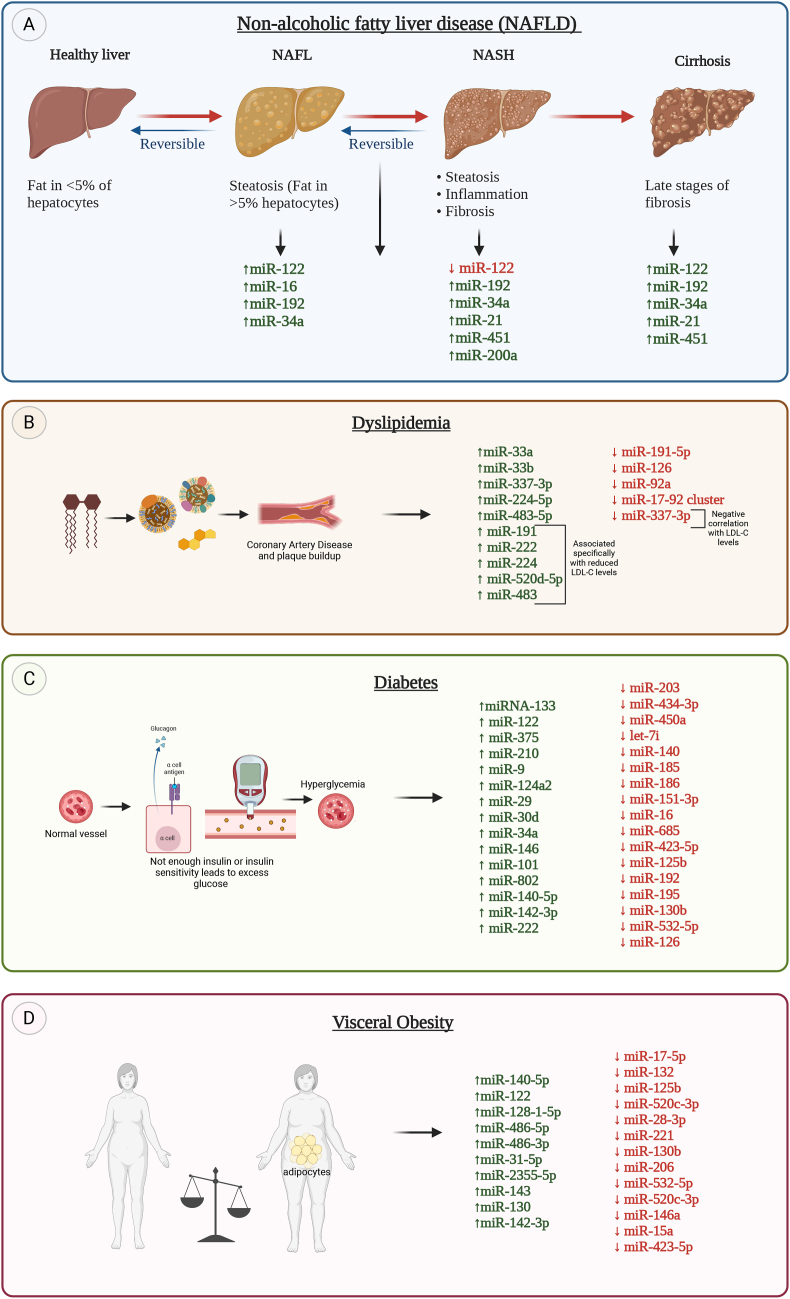
**(A)** Differential expression of circulating miRNAs during progression of fatty liver disease. **(B)** Upregulated and downregulated miRNAs in dyslipidemia and progression of coronary artery disease. **(C)** Cell-free miRNA expression in diabetes. **(D)** Expression of cell-free miRNAs in the context of visceral obesity. Green color and upwards pointing arrows indicate increased expression while red color and downwards facing arrows indicate lower expression.

Metabolic diseases encompass a range of conditions characterized by dysregulation in energy utilization and storage. These conditions often involve disturbances in glucose and lipid metabolism, leading to states with elevated cholesterol such as dyslipidemia. Furthermore, as individuals age, there is a notable increase in the prevalence of metabolic diseases, marked by complex alterations in various physiological processes. One prominent aspect is the development of insulin resistance, where cells become less responsive to insulin, impairing glucose homeostasis. Age-related changes in steroid hormone levels, including alterations in cortisol and sex hormones, also contribute to metabolic shifts. These changes collectively contribute to an elevated risk of conditions such as type 2 diabetes, cardiovascular diseases, and metabolic syndrome among the aging population. In the context of liquid biopsy or other diagnostic approaches, both miRNAs and other forms of cfRNA can be analyzed for potential biomarkers associated with such diseases. Notably, individual profiles of cfRNA have been explored in conditions such as diabetes, fatty liver disease, and more, offering insights into the molecular underpinnings of these disorders (Figure 2 and Table 1).

**Table 1:** Changes in cfRNAs in NAFLD, dyslipidemia, obesity and diabetes.

	Preclinical and clinical observations	References
NAFLD/Liver Fibrosis/Hepatic Damage		
miR-122	Elevated in CHC, liver steatosis, fibrosis, and NAFLD patients, downregulated in NASH compared to simple steatosis, overall regulated in NASH	[26,[29], [30], [31], [32], [33], [34], [35]]
miR-16	Elevated in liver fibrosis, CHC and NAFLD patients	[33,35,39]
miR-192	Correlates with the progression of NAFLD and elevated in liver fibrosis and steatosis	[29,30,35,39]
miR-34a	Elevated in CHC, liver steatosis, fibrosis, NASH and NAFLD	[28,30,31,35,38,39,41]
miR-451	Elevated in liver steatosis and fibrosis	[31]
miR-21	Elevated in liver steatosis and fibrosis, and NASH	[28,35,38]
miR-200a	Strongly correlated with fibrosis and associated with insulin resistance	[30]
hsa-miR-122-5p and hsa-miR-885-5p	Elevated in fatty liver disease	[35,42]
Dyslipidemia		
miR-33a and miR-33b	Upregulated in children with familial hypercholesterolemia and positively correlated with lipid and inflammatory markers	[57]
miR-337-3p, miR-337-5p	Negative correlation with serum LDL-C levels and upregulated in CAD patients	[59,60]
miR-191-5p	Reduced expression in CAD patients and increased expression after PCSK9 treatment	[56]
miR-224-5p	Elevated expression in CAD patients and reduced expression after PCSK9 treatment	[56]
miR-483-5p	Elevated expression in CAD patients and involved in regulating serum PCSK9 levels in CVD	[56]
miR-191, miR-222, miR-224, miR-520d-5p, miR-483	Associated with reduced LDL-C levels	[56]
miR-486 and miR-92a	Increased in hyperlipidemic hamsters	[61]
let-7a, miR-126, miR-21, miR-26a	Significantly elevated levels in hyperlipidemic rats	[62]
miR-29a and miR-145-5p	Decreased levels in hyperlipidemic rats	[62,63]
miR-126, miR-92a, or the miR-17-92 cluster	Downregulated in patients with CAD and in patients on statin therapy	[64]
Diabetes		
miRNA-133	Persistent increased levels across all stages of T2D in rats	[12,66]
miR-203	Persistent decreased levels across all stages of T2D in rats	[12,66]
miR-122	Increased during β cell failure in T2D and in patients with metabolic syndrome. Reduced in treatment with statins	[12,66,76]
miR-434-3p	Decreased during β cell failure in T2D in rats	[12,66]
miR-450a	Decreased during β cell failure and late stage T2D in rats	[12,66]
miR-375	Increased in late-stage T2D and prior to onset of hyperglycemia with high doses of STZ in rats. Significantly increased in new-onset T2D	[12,67,68,70]
miR-210	Increased in late-stage T2D	[66]
let-7i, miR-140, miR-185, miR-186, miR-151-3p, miR-16, miR-685	Decreased in late-stage T2D	[12,69]
miR-9, miR-124a2, miR-29, miR-30d, miR-34a, miR-146	Increased in new-onset T2D	[12,69]
miR-101 and miR-802	Significantly higher in T2D	[12,70]
miR-140-5p, miR-142-3p, and miR-222	Increased in T2D	[71]
miR-423-5p, miR-125b, miR-192, miR-195, miR-130b, miR-532-5p, miR-126	Decrease in T2D	[71]
lncRNA-NR_033515	Increased in DN	[72]
Obesity		
miR-192, miR-27a-3p, miR-27b-3p	Increased in the plasma exosomes of obese mice	[82]
miR-17-5p and miR-132	Significant decrease in the omental fat and whole blood of obese patients	[86,87]
miR-140-5p	Increased concentration in morbidly obese patients	[87,88]
miR-125b and miR-520c-3p	Decreased concentration in morbidly obese patients	[87,88]
miR-122	Increased in the plasma exosomes of obese mice. Increased in patients with higher hepatic fat at baseline and reduced after diet and physical activity interventions.	[64,68,89]
miR-128-1-5p	Elevated in patients with increased insulin resistance, waist circumference, total body fat mass, and resting energy expenditure. Reduced weight loss intervention	[90]
miR-138, miR-15b, miR-376a	Deregulated in patients with obesity	[91]
miR-28-3p	Decreased in children with prepubertal obesity	[93]
miR-221	Decreased concentration in morbidly obese patients and obese prepubertal children	[87,88,93]
miR-486-5p, miR-486-3p	Increased in children with prepubertal obesity	[93]
miR-130b	Decreased concentration in morbidly obese patients and increased in obese prepubertal children	[87,88,93]
miR-31-5p and miR-2355-5p	Upregulated in overweight/obese prepubertal children	[95]
miR-206	Downregulated in overweight/obese prepubertal children	[95]
miR-21, miR-27b, miR-29a, miR-150, and miR-223	Significant postitive correlation with BMI	[94]
miR-140-5p, miR-222, miR-143, miR-130	Increased in morbidly obese adolescents	[96]
miR-142-3p	Increased concentration in obese patients including adolescents	[87,88,93,96]
miR-532-5p	Decreased concentration in morbidly obese patients including adolescents	[87,88,96]
miR-520c-3p and miR-146a	Decreased in morbidly obese adolescents	[96]
miR-15a	Decreased concentration in morbidly obese patients including adolescents	[87,88,96]
miR-423-5p	Decreased in morbidly obese patients including adolescents. Increased in obese prepubertal children	[87,88,96]

### 2.1 Fatty liver disease, hepatic fibrosis and cfRNA signatures

Nonalcoholic fatty liver disease (NAFLD), sometimes also referred to as metabolic dysfunction-associated steatotic liver disease (MASLD) in recent nomenclature [24], is defined by hepatic steatosis, linked to metabolic risk factors, and histologically classified into nonalcoholic fatty liver (NAFL) marked by steatosis, and nonalcoholic steatohepatitis (NASH) characterized by steatosis, inflammation, hepatocyte injury, and potentially fibrosis. The diagnosis excludes secondary causes of hepatic fat accumulation [25]. The escalating prevalence (32% of the adult population globally) of nonalcoholic fatty liver disease (NAFLD) poses a substantial health challenge, with associated metabolic complications, cardiovascular risk, and mental health implications [26]. NAFLD is often linked to metabolic risk factors such as obesity, diabetes mellitus, and dyslipidemia [25]. Liver fibrosis can be the consequence of prolonged hepatic damage, characterized by excessive extracellular matrix synthesis and accumulation. It can be the result of NAFLD and NASH but it can be caused by other hepatotoxic insults such as chemicals, viruses etc [27]. If left unaddressed, fibrotic processes can ultimately lead to cirrhosis. Hepatic fibrosis involves the abnormal production and accumulation of extracellular matrix proteins and clinically the elevation of hepatic enzymes such as AST and ALT are useful. However, they sometimes lack specificity and sensitivity or their disturbance may become apparent once a liver damage is already established. Plasma albumin mRNA has been described in the past as a marker of liver pathologies such as cirrhosis or virus-induced hepatic inflammation [28].

Transitioning to the focus on cfRNA signatures, emerging research investigates the molecular intricacies of NAFLD. cfRNA signatures hold promise as diagnostic and prognostic indicators, offering insights into the underlying pathophysiological mechanisms of NAFLD (Figure 2A). Multiple studies have found elevated circulating and hepatic miR-122 levels in NAFLD [[29], [30], [31], [32], [33], [34], [35]] as it is a very abundant microRNA (miRNA) in the liver [29,35], playing roles in diverse hepatic functions (lipid metabolism, iron homeostasis) and hepatic differentiation [36]. It has also been found that circulating miR-122, miR-34a and miR-16 levels are elevated in chronic hepatitis C (CHC) and NAFLD patients with the first two correlating with disease severity, liver enzymes, fibrosis stage, and inflammation activity, suggesting their potential as noninvasive diagnostic and histological markers for liver diseases [35,37]. Utilizing a two-stage strategy encompassing global serum miRNA profiling and liver expression analysis, it was uncovered that elevated miRNAs, notably miR-122 and miR-192, correlate with the progression of NAFLD, with miR-122 exhibiting noteworthy downregulation in NASH compared to simple steatosis, and exerting an impact on alanine aminotransferase activity [30,35]. miR-34a and miR-122 levels were significantly increased in NASH patients, positively correlating with stages of inflammation and fibrosis and miR-21 exhibited increased levels in NASH patients compared to healthy controls and NAFLD patients [35,38]. Similar findings were observed in patients diagnosed with NAFLD, where those with more severe liver steatosis exhibited higher levels of miR-122, suggesting that miR-122 could be used as a simplified screening marker for NAFLD. Additionally, participants also had elevated levels of miR-21, miR-34a, and miR-451 [31,35]. Similarly, in a study of 132 subjects with NAFLD, miR-34a, miR-122, miR-192, and miR-200a were strongly correlated with fibrosis [33]. Further, miR-34a had the strongest predictive value for fibrosis stages, while miR-200a was specifically associated with the TM6SF2 E167K variant and insulin resistance [33]. miR-34 was also found to have a twofold increase in NAFLD compared to chronic hepatitis B. The study also showed that serum miR-122, miR-192, and miR-34a levels correlated with steatosis and inflammatory activity while miR-16 was only associated with fibrosis [35,39]. These studies altogether show similar trends in the miRNAs associated with NAFLD while revealing distinct relationships of these circulating miRNAs with NAFLD severity and pathogenic factors.

A study by Miyaaki et al. investigated the relationship between the liver-enriched miR-122 expression levels in the liver and serum of patients with NAFLD. The findings revealed a significant correlation between hepatic and serum miR-122 levels, with lower hepatic miR-122 associated with severe steatosis and lower fibrosis levels [29,35]. A similar study found strong associations between the serum miR-122 ratio (ratio of levels at the second biopsy to the first during the follow-up of patients) and changes in histopathological scores, including steatosis, lobular inflammation, and stage, with significantly decreased miR-122 levels in the second biopsy in patients with improved histopathological scores [35,40]. In addition to miR-122, miR-34a also showed significant elevation in NAFLD patients compared to healthy controls, however, their levels did not correlate with histological features of NAFLD [35,41]. Elevated levels of hsa-miR-122-5p and hsa-miR-885-5p were also linked to fatty liver and slightly enhanced fatty liver detection beyond established risk factors when adjusted for age, sex, and BMI [35,42]. This study suggests that serum miR-122 could serve as a valuable predictive marker for liver fibrosis in NAFLD patients and it dynamically changes regarding the evolution of the disease. Thus, it is important to evaluate its increased or decreased levels depending on the baseline status of the patient, the stage of the disease and the therapeutic intervention (if any). Figure 2A summarizes most of the findings regarding the expression of cfRNAs in the progression of fatty liver disease.

An evaluation of cfmRNAs in patients with NAFLD and fibrosis revealed notable differences compared to healthy individuals. The comparison showed 1527 upregulated and 971 downregulated genes in NAFLD patients, which are involved in processes such as immune system response, metabolic processes, and changes in cellular component organization or biogenesis, all commonly associated with chronic inflammation and fibrosis [43]. Additionally, 134 fibrosis-associated genes were identified in patients with NAFLD [43]. Furthermore, a cf-mRNA classifier was shown to be able to predict liver fibrosis stage in an independent cohort. This demonstrates the potential for a cf-mRNA-based NAFLD fibrosis classifier, offering a clinically noninvasive method for fibrosis staging [43].

Certain circRNAs functioned as promoters of hepatic fibrosis while others acted as inhibitors. In irradiated human hepatic stellate cell (HSC) line LX2, the circRSF1 was found to be upregulated and predicted to have binding sites for miR-146a-5p. Subsequent experiments confirmed the direct interaction between circRSF1 and miR-146a-5p by acting as a sponge for miR-146a-5p, inhibiting its activity. This resulted in enhanced cell viability, inflammation, and a fibrotic phenotype [44]. Circ-PWWP2A was found to sponge miR-203 and miR-223, promoting HSC activation by increasing Fstl1 and TLR4 expression, respectively. Inhibiting circ-PWWP2A alleviated hepatic fibrosis in vivo, suggesting that circ-PWWP2A serves as a common downstream mediator of TGF-β and LPS in HSC activation and fibrogenesis [45]. Overexpression of circFBXW4 was found to attenuate liver fibrogenesis and inflammation by targeting miR-18b-3p to regulate FBXW7 expression [46]. Similarly, circCREBBP was found to be down-regulated in carbon tetrachloride-induced hepatic fibrosis. Overexpressing circCREBBP reduced liver damage and fibrosis *in vivo* and inhibited HSC activation and proliferation by acting as a sponge for hsa-miR-1291, promoting LEFTY2 expression [47].

LncRNA NEAT1 and GRIA3 were upregulated in NAFLD patients and thought to suppress miR-212-5p concentration, promoting lipid accumulation [48]. Similarly lncARSR was found to promote hepatic lipogenesis via upregulation of the Akt/SREBP-1c pathway, contributing to hepatic steatosis in NAFLD [49]. Other IncRNA including lncRNA RABGAPILDT-206, Inc-SPARCL1-1:2, Inc PVT1, Inc HCG18 also had increased expression in patients with NAFLD through various intracellular pathways [50].

### 2.2 Dyslipidemia, atherosclerosis and cfRNA signatures

Hyperlipidemia poses a significant risk for vascular endothelial injury, contributing to the development of atherosclerosis and other cardiovascular diseases. Atherosclerotic cardiovascular disease may lead to ischemic heart disease and ischemic stroke, ranking as the leading and fifth causes of death worldwide, respectively [51]. Even though a variety of clinical markers such as levels of total cholesterol, HDL cholesterol, LDL-cholesterol, triglycerides, apoA lipoprotein, hs-CRP are being used to evaluate the cardiovascular risk, there is always need for more precise biomarkers that reflect an actual ongoing process of atherosclerosis so as to alert clinicians to intensify a cholesterol-lowering treatment, to ensure a much closer follow-up, and also to monitor the efficiency of a treatment with relevance to the modulation of the cardiovascular risk [52].

Proprotein convertase subtilisin kexin 9 (PCSK9) that regulates the levels of the LDL receptor [53] has attracted the interest in research in the field of lipidology not only as target for treatment (PCSK9 inhibitors) but also as a marker for atherosclerosis [54,55]. In this context, research has also been focused on miRNAs that can directly or indirectly affect the expression of PCSK9 and thus serve as circulating markers. In patients with stable coronary artery disease (CAD) and elevated lipoprotein a in serum showed a notable decrease in miR-191-5p expression and elevated miR-224-5p and miR-483-5p expression levels in patients compared to control subjects, with miR-483-5p expression significantly predicting baseline serum PCSK9 levels [56]. Specifically, the circulating levels of miR-483 were found to be inversely correlated with serum levels of total cholesterol and LDL cholesterol, as miR-483 targets PCSK9 mRNA [56].

The expression of circulating miR-33a and miR-33b was investigated to determine if it is altered in children with familial hypercholesterolemia (FH) [57]. miR-33 is known to play roles in cardiac remodeling, in lipid raft cholesterol content in fibroblasts and in adaptive fibrotic responses [58]. Results revealed a significant up-regulation of miR-33a and miR-33b in hypercholesterolemic children with positive correlations with various lipid and inflammatory markers [57]. Other studies demonstrated that miR-337-3p levels were notably reduced in various hyperlipidemic mouse models, showing a consistent negative correlation with serum LDL-C levels [59]. miR-337-5p levels have been shown to be elevated in patients with stable angina [60]. *In vitro* and *in vivo* experiments confirmed that miR-337-3p plays a role in improving serum LDL-C by interacting with both the PCSK9 3′UTR and promoter, leading to the inhibition of PCSK9 translation and transcription [60].

Further studies showed that hyperlipidemic hamsters exhibited elevated levels of liver (2.8-fold) and plasma (2-fold) miR-486, and increased miR-92a (2.8-fold and 1.8-fold, respectively) compared to normolipidemic hamsters [61]. Following a 2-week treatment with lock-nucleic acid inhibitors for either miR-486 or miR-92a, liver and plasma cholesterol levels notably decreased (23% and 17.5% for anti-miR-486, 16% and 22% for miR-92a inhibition) [61]. Plasma levels of let-7a, miR-126, miR-21, and miR-26a were also significantly elevated in hyperlipidemic rats at 30 and 50 days after intraperitoneal injection of vitamin D3 combined with a high-fat diet. Conversely, the plasma level of miR-29a was notably decreased, suggesting its potential as an early (>20 days) diagnostic biomarker for endothelial injury-related diseases [62]. Other hyperlipidemia rat models exhibited decreased levels of miR-145-5p which regulates lipid metabolism and M2 macrophage polarization [63]. In patients with CAD, highly expressed miRNAs such as miR-126, miR-92a, or the miR-17-92 cluster originating from the vessel walls and inflammatory cells were also shown to have decreased expression in the blood. Statin therapy also revealed a tendency towards decreased levels of miR-17, miR-92a, and miR-126 in comparison to those without statin treatment [64].

Figure 2B summarizes some of the known associations between cfRNAs and dyslipidemia.

### 2.3 Diabetes and cfRNA signatures

Type 2 diabetes (T2D) is characterized by increased peripheral insulin resistance and a relative insufficiency of insulin secretion so as to overcome the insulin resistance. Type 1 diabetes (T1D) is the result of β cell failure mainly due to autoimmune destruction. Diabetes is associated with cardiovascular and renal complications in the long-term. Besides the circulating glucose levels and auto-antibodies in type 1 diabetes, more markers are needed so as to predict the development of diabetes and/or its complications [65].

A preclinical study examined the changes in miRNAs during the development and progression of T2D from six to seventeen weeks in Zucker diabetic fatty rats with a defective leptin pathway, resulting in various metabolic diseases including T2D [66]. The results of this study identified changes in circulating miRNAs over time. During initial hyperinsulinemia, miRNA-133 levels increased, while miR-203 levels decreased. As β cell failure occurred, miR-133a remained elevated, miR-122 increased, and miR-203, miR-450a, and miR-434-3p decreased. In late-stage diabetes, there was increased levels of miR-375, miR-210, and miR-133a, and decreased levels of let-7i, miR-140, miR-450a, miR-185, miR-186, miR-151-3p, miR-203, miR-16, and miR-685. Notably, miR-133a and miR-203, exhibited a persistent increased and decreased alteration respectively across all disease stages. These results suggest that certain miRNAs may be useful as biomarkers in tracking T2D progression and treatment response [12,66]. However, these miRNAs participate in various and diverse processes in different tissues and these changes may be the the results of metabolic disturbances that have already occurred.

Similarly, a study measuring miR-375 levels in plasma of mice treated with streptozotocin (STZ) (toxic to islet β cells) showed significantly increased circulating miR-375 levels before the onset of hyperglycemia with high doses of STZ [67]. In non-obese diabetic (NOD) mice, a mouse model of autoimmune diabetes, resembling T1D, plasma miR-375 levels were significantly elevated two weeks prior to the onset of diabetes. Supporting *in vitro* studies with employing cytotoxic insults to β-cells also showed increased extracellular miR-375 levels which was mitigated with the use of cell-death inhibitors. This suggests that miR-375 could potentially serve as a marker for β-cell death and a predictor of diabetes [12,67] even though it has to be used with caution as only a small proportion of circulating miR-375 appears to come from β cells [68].

In a clinical study, expression profiles of seven circulating miRNAs related with diabetes of 56 patients at different stages of T2D were analyzed, namely miR-9, miR-124a2, miR-375, miR-29, miR-30d, miR-34a, and miR-146 [69]. Results showed that miRNAs negatively regulated insulin-related processes. In patients with new-onset T2D, all seven miRNAs showed a significantly elevated expression compared to those with normal glucose tolerance and a significantly elevated expression in 5/7 miRNAs when compared to prediabetics. However, in the prediabetic stage, the expression patterns closely resemble those with normal glucose tolerance which suggest that the miRNA profiles do not change substantially during this stage [12,69]. Another clinical study also showed that serum concentrations of miR-101, miR-375, and miR-802 were significantly higher in T2D patients compared to normal glucose tolerance subjects. Stepwise regression analysis identified HbA1c as an independent predictor of miR-101, while eGFR, HbA1c, and HDL-C values were significant determinants of serum miR-802 levels [12,70]. Further, research showed that in T2D patients, miR-140-5p, miR-142-3p, and miR-222 levels increased, while miR-423-5p, miR-125b, miR-192, miR-195, miR-130b, miR-532-5p, and miR-126 levels decreased. Four miRNAs (miR-140-5p, miR-423-5p, miR-195, and miR-126) demonstrated high specificity for T2D with an accuracy of 89.2%. Treatment with metformin induced significant changes in the levels of miR-192, miR-140-5p, and miR-222, which corresponded to decreases in fasting glucose and HbA1c [71].

A significant increase in lncRNA-NR_033515 expression was detected in the serum of diabetic nephropathy (DN) patients, correlating with different disease stages and positively associated with diagnostic markers (KIM-1 and NGAL) [72]. NR_033515 was found to modulate P38, ASK1, Fibronectin, α-SMA, E-cadherin, and Vimentin expressions through miR-743b-5p, suggesting a potential role for NR_033515 in DN's proliferation, fibrogenesis, and epithelial–mesenchymal transition. These findings propose NR_033515 as a promising diagnostic and therapeutic target for managing DN [72]. As this study compared healthy patients versus diabetic ones with diabetic nephropathy, it could also be possible that LncRNA-NR_033515 can be a marker of diabetes *per se*. Examination of US veterans identified that decreased lncRNA GAS5 levels, which regulates cell growth, proliferation, and survival, was associated with a higher risk of T2D [73]. Hsa_circ_0054633 has also been extensively studied, showing increased expression in response to elevated glucose levels in DM and exhibiting protective effects against high glucose-induced endothelial cell dysfunction by inhibiting miRNA-218 expression [74]. CircANKRD36 was also positively correlated with inflammatory markers in T2D patients and interacts with various miRNAs involved in T2D and inflammation-associated pathways [75].

Furthermore, the correlation between miRNA and metabolic syndrome was highlighted by findings showing that participants with metabolic syndrome had circulating miR-122 levels 160% higher than those without the condition [76]. Similarly, participants diagnosed with T2D had circulating miR-122 levels that were 214% higher compared to those without T2D. These associations were consistent regardless of the degree of adiposity. Circulating miR-122 levels did not differ significantly between individuals with and without a history of cardiovascular disease. Treatment with statins was found to reduce both lipoprotein and miR-122 release from the liver by possibly inhibiting protein prenylation in cholesterol synthesis, thus decreasing the secretion of hepatic exosomes, where miR-122 is abundant. Circulating miR-122 was undetectable in serum depleted exosomes. Reduced miR-122 was also seen in mice treated with atorvastatin, further confirming its role as a marker in lipid metabolism [76].

It appears that the majority of existing studies regarding the expression of cfRNAs in diabetes are mostly targeted in assessing the expression of miRNAs by specifically measuring some of these species that are related with insulin resistance, β cell mass and metabolic dysfunction in general. Figure 2C summarizes some of the existing data and it seems that more research is warranted by using a more unbiased approach of larger sample sizes that study all cell-free RNA species, not being limited to miRNAs only.

### 2.4 Obesity and cfRNA signatures

The escalating prevalence of obesity poses a significant health challenge, with associated metabolic complications, cardiovascular risks, and mental health implications [77]. According to WHO 39% of adults aged 18 years and over were overweight in 2016, and 13% were obese. The USA obesity prevalence was 41.9% in 2017–March 2020, emphasizing the critical need for systematic and decisive approaches [78].

In a preclinical mouse study on obesity, toll-like receptors (TLR) were crucial for immune response activation. Plasma from obese mice showed TLR3 and TLR8 activation, blocked by specific inhibitors, confirming ligand presence [79]. Depleting ssRNA hindered TLR3 activation, suggesting potential double-stranded structures. The study found a ∼40% increase in total cfRNA in the plasma of obese mice without a significant change in concentration from changes in diet. After treatment with PAMAM generation three [80] to promote an anti-inflammatory and anti-obesity effect, cfRNA in the plasma stabilized after eight weeks with a significant reduction in TLR3 activation. This suggests that cfRNA in obese subjects could be a potential marker of chronic inflammation [79].

Mice lacking the miRNA-processing enzyme Dicer in adipose tissue, and humans with lipodystrophy exhibit reduced levels of circulating exosomal miRNAs. Transplantation of adipose tissue, especially brown, restores miRNA levels, improving glucose tolerance and reducing hepatic fibroblast growth factor 21 (Fgf21) mRNA and circulating FGF21, suggesting that adipose tissue serves as a source of circulating exosomal miRNAs [81]. Obesity was also shown to alter the miRNA profile of plasma exosomes in mice, leading to increased levels of miR-122, miR-192, miR-27a-3p, and miR-27b-3p. Treatment of lean mice with exosomes containing these obesity-associated miRNAs induced glucose intolerance, insulin resistance, central obesity, and hepatic steatosis, emphasizing the central role of exosomal miRNAs in the development of metabolic abnormalities associated with obesity [82].

Several circRNAs and lncRNAs have also been implicated in adipogenesis, a process central to the development of obesity. In subcutaneous adipose tissues, circRNA_26852 was found to be up-regulated, while circRNA_15067, circRNA_23437, circRNA_14707, circRNA_11897, lncRNA-p19461, lncRNA-p5549, and lncRNA-p21015 were down-regulated [74,83,84]. CircRNA_26852 and circRNA_11897 targeted genes involved in adipocyte differentiation and lipid metabolism, with circRNA_11897 regulating miRNA-27a and miRNA-27b-3p through a competing endogenous RNA mechanism [74,84]. In high-fat diet-induced obese mice, 3203 lncRNAs were detected with a significant downregulation in lncRNA 1810019D21Rik, contributing to beta-cell dysfunction [85].

Clinical studies have also shown significantly decreased miR-17-5p and miR-132 levels in both omental fat and whole blood of obese individuals compared to non obese counterparts. The expression of miR-17-5p also exhibited a notable negative correlation with BMI in obese patients [86,87]. Another study examining specific plasma miRNA signatures in morbidly obese patients, linked nine circulating miRNAs to fat mass measures. miR-140-5p and miR-142-3p show increased concentrations, while miR-532-5p, miR-125b, miR-130b, miR-221, miR-15a, miR-520c-3p, and miR-423-5p exhibit decreased concentrations in morbidly obese patients. Surgically-induced weight loss modulated 14 circulating miRNAs, including downregulation of miR-140-5p and miR-122 and upregulation of miR-221 and miR-199a-3p [87,88]. Increased circulating miR-122 was also associated with higher hepatic fat at baseline and a lower reduction in hepatic fat percentage in response to 18-month diet and physical activity interventions [89]. High levels of miR-128-1-5p were associated with increased insulin resistance, waist circumference, total body fat mass, and resting energy expenditure, while changes in miR-128-1-5p levels during interventions were linked to improvements in weight loss outcomes [90].

In one study, serum samples from individuals with T2D, obesity, both conditions and healthy controls were analyzed and found that three miRNAs (miR-138, miR-15b, and miR-376a) were potential predictive biomarkers for obesity, with miR-138 or miR-376a distinguishing obese patients alone and the combination of miR-503 and miR-138 was effective in distinguishing diabetic from obese diabetic patients [91].

When examining the expression of fetal cfRNA in obese women, results showed that mid-trimester amniotic fluid from obese pregnant women had significant differential regulation of 205 genes, including up-regulation of Apolipoprotein D, a central nervous system gene, and down-regulation of apoptotic cell death, with predicted activation of pro-estrogenic and pro-inflammatory pathways cia activation of FOS, and STAT3 transcriptional regulators estrogen receptors [92]. In prepubertal obese patients, 15 miRNAs were significantly deregulated, including decreased miR-221 and miR-28-3p and increased concentrations of miR-486-5p, miR-486-3p, miR-142-3p, miR-130b, and miR-423-5p. These miRNAs were associated with various obesity-related measures and may help identify prepubertal obese children at risk of metabolic abnormalities [93]. A study conducted in obese children also found a statistically significant correlation between BMI and five miRNAs (miR-21, miR-27b, miR-29a, miR-150, and miR-223), with miR-29a exhibiting the most robust association [94]. Similarly, research assessing circulating miRNAs in overweight/obese prepubertal children showed a twofold upregulation of miR-31-5p, a threefold upregulation of miR-2355-5p, and a 0.5-fold downregulation of miR-206 compared to normal weight children [95]. Morbidly obese adolescents also exhibited altered concentrations of at least 10 circulating miRNAs, including increased levels of miR-142-3p, miR-140-5p, miR-222, miR-143, and miR-130, and decreased levels of miR-532-5p, miR-423-5p, miR-520c-3p, miR-146a, and miR-15a, which were strongly linked to BMI, waist to height ratio (WHtR), adipokines, and other metabolic syndrome-related biomarkers [96]. A study conducted in obese children also found a statistically significant correlation between BMI and five miRNAs (miR-21, miR-27b, miR-29a, miR-150, and miR-223), with miR-29a exhibiting the most robust association [34].

Figure 2D summarizes some of the existing data regarding the expression of cfRNAs in obesity. Some of these circulating miRNAs regulate basic processes in adipose tissue such as adipocyte differentiation, adipose tissue expansion and inflammation [97] and thus they may be derived from this tissue. Further analyses are warranted so as to identify the tissue of origin of these cfRNAs and their roles, expand the analyses in all types of cfRNAs, not only in miRNAs, and potentially create algorithms that can predict the evolution of obesity and its related comorbidities.

### 2.5 cfRNA profiles and bariatric surgery

**Figure 3:**
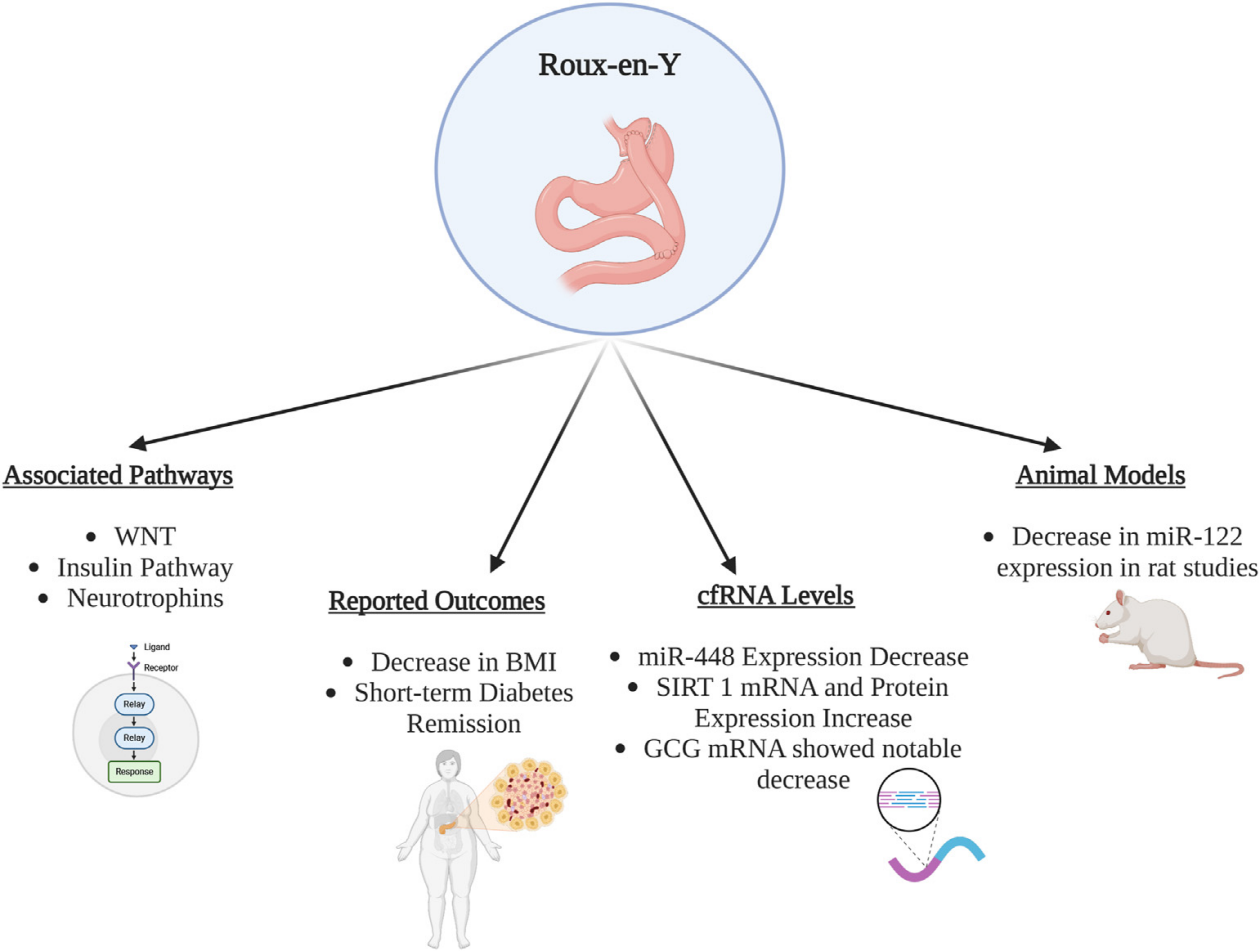
Effects of bariatric surgery (RYGB) on metabolic measures and cfRNA profiles.

Bariatric surgery, acknowledged as an effective intervention for obesity and its related comorbidities, brings about substantial alterations in the transcriptome, with a notable impact on microRNA expression (Figure 3). A systematic review and meta-analysis, encompassing 17 studies with animal models and humans, revealed that 14 microRNAs were consistently changed after surgery [98]. These microRNAs, including hsa-miR-93-5p, hsa-miR-106b-5p, and hsa-miR-7-5p, offer insights into potential pathways implicated in the beneficial effects of bariatric surgery on weight loss and obesity-related conditions [98]. However, no associations were made between the expressions of these miRNAs and the body weight loss and the evolution of related comorbidities post-bariatric surgery.

Several other studies have been conducted that look at outcomes via the study of circulating miRNAs post bariatric procedures [88,[98], [99], [100], [101], [102], [103], [104]]. In a longitudinal study, individuals underwent bariatric surgery, specifically Roux-en-Y gastric bypass (RYGB), to evaluate the temporal impact on circulating miRNA expression profiles [99]. Utilizing Exiqon's optimized panel and miRCURY locked nucleic acid plasma/serum PCR, the study revealed a time-dependent alteration in the circulating microRNAome postoperatively. Notably, 48 circulating miRNAs exhibited significant differences, correlating with pathways associated with metabolic regulation and rescue, as well as demonstrating correlations with BMI, percentage of excess weight loss, and fasting blood glucose levels [99]. In another study, following RYGB surgery in patients with obesity and diabetes, a significant decrease in BMI (3.8 kg/m^2^) was observed at day 21 postoperatively, with 62% experiencing short-term diabetes remission [100]. Seven miRNAs, including miR-7-5p and miR-320c, exhibited significant changes post-surgery, associated with pathways related to diabetes, insulin resistance, and intestinal functions [100]. Similarly, research identified 72 differentially expressed exosomal miRNAs in patients with obesity compared to healthy volunteers, and post-bariatric surgery, 41 miRNAs exhibited altered expression, including nine surgery-responsive miRNAs associated with pathways such as WNT, insulin, and neurotrophins [105]. lncRNA H19 levels specifically showed decreased expression in subcutaneous adipose tissue for obese patients one year post-bariatric surgery and also correlated positively with excess weight loss and negatively with initial BMI [106].

Specific exosomal miRNAs associated with insulin signaling, derived from obese adipose tissue have been examined in the context of gastric bypass surgery [107]. Results indicated that one year post-surgery, 168 surgery-responsive miRNAs, including those correlated with changes in insulin resistance and branched chain amino acids, were identified, highlighting a targeted modulation of miRNA expression in relation to improved insulin sensitivity (miR-1227-3p, miR-4691-5p and miR-219a-5p upregulation) [107]. Additionally, the impact of RYGB on serum miRNA profiles in T2D patients with different body mass indexes (BMI) and insulin levels was also investigated [103]. RYGB induced significant changes in specific miRNA expression (namely, downregulated expression of let-7, miR-24, miR-24-23a/b, miR-24-93, miR-24-26a, miR-24-151-3p, miR-24-425, miR-24-151-5p, miR-24-146a, and miR-24-103a, and upregulated expression of miR-4787-5p and miR-24-1281) suggesting roles for miRNAs in ameliorating insulin sensitivity [103]. Some of these miRNAs were found to change in adipose tissue following laparoscopic RYGB, indicating the adipose tissue as a potential source of these miRNAs that play roles in insulin signaling, inflammation and adipocyte differentiation [108]. Furthermore, Wang et al. assessed the expression and prognostic significance of peripheral blood miR-448 and its target gene *SIRT1* in patients with obesity and T2DM undergoing laparoscopic bariatric surgery, revealing that miR-448 expression decreased while SIRT1 mRNA and protein expression increased, particularly in the effective treatment group [109]. The findings suggest that miR-448 and SIRT1 can serve as outcome indicators post-surgery in obese T2DM patients.

After undergoing bariatric surgery, individuals witness marked enhancements in metabolic well-being, as demonstrated by notable reductions in body weight, BMI, and the reversal of diabetes mellitus [110]. These outcomes suggest an improvement in the function and health of pancreatic β cells. Given the inherent challenges in directly assessing molecular changes within organs like the pancreas in living patients, the potential utility of liquid biopsies becomes apparent [111]. Whye et al. demonstrated that GCG circulating cfmRNA levels displayed notable associations with common markers of metabolic amelioration post-bariatric surgery, including hemoglobin A1c levels (R: −0.41, p-value: 0.0039) and the percentage of excess weight loss (R: 0.29, p-value: 0.046) [111,112]. Targeting circulating mRNA transcripts associated with pancreatic health, liquid biopsies offer a promising non-invasive avenue to assess molecular alterations following bariatric surgery [111]. Building on their success in probing molecular changes in challenging-to-reach tissues like the brain, liquid biopsies may serve as valuable tools for examining ongoing cellular transformations contributing to enhanced pancreatic health post-surgery [9,111,112].

Animal models have also been utilized to study the miRNA phenotypic relationship pre- and post-bariatric surgery. A study on rats investigated the impact of RYGB on miRNA expression, particularly focusing on miR-122, in male Sprague–Dawley rats [113]. Findings revealed significant changes in miR-122 expression in the hypothalamus, heart, and liver, suggesting a regulatory role of RYGB in modulating miR-122 levels, potentially influencing the activities of the metabolic regulator AMP-activated protein kinase [113]. Another rat study looked at the mechanisms underlying sustained weight loss and type 2 diabetes remission post-RYGB surgery [104]. Results revealed a distinctive response of miRNAs to RYGB, specifically a significant decrease in circulating miRNA-122 levels, suggesting their involvement in key signaling pathways related to G protein signaling, neurodegeneration, inflammation, and growth and apoptosis responses. Studies also found an upregulation in the expression of 232 lncRNA and downregulation in 69 lncRNA after bariatric surgery in high-fat diet-induced diabetic mice [114]. The findings depict the role of miRNAs in mediating responses within metabolic pathways, particularly during RYGB-induced therapeutic effects [104].

## 3 Conclusions

Exploring the cfRNA profiles in obese individuals with metabolic complications, including diabetes, fatty liver, dyslipidemia, and heart disease, holds promise for uncovering insights into the cellular processes underpinning these various phenotypes as well as for developing effective biomarkers to improve the quality of life of these patients. A wide variety of cfRNAs have been detected in different studies with the minority of them overlapping among different studies. The majority of the cfRNAs described are miRNAs possibly due to the fact that they are stabler in circulation [115] and were easier to study in the past due to already developed assays for them. Relatively recent advances in the technologies for the preservation of various cfRNAs species in the circulation [18] and the evolution and affordability of next-generation sequencing [17] can lead to a more thorough analysis of all circulating RNA species.

In the future, when conducting such studies, it is important to characterize in depth the metabolic phenotype of the subjects as some of the observed changes in cfRNAs may not be due to the presence of the metabolic disease being investigated but due to the presence of another complication or a drug treatment. Identification of the tissue of origin for differentially expressed cfRNAs could drive further investigations into the cellular and tissue-level mechanisms and possibly inter-organ crosstalk contributing to metabolic complications. Moreover, the distinctive cfRNA patterns may serve as early biomarkers for detecting metabolic complications associated with obesity, offering opportunities for early intervention to prevent or slow down conditions such as fatty liver, NASH, hepatic fibrosis and diabetes. cfRNA can also be used as a detection marker as a means to prevent carcinogenesis in fatty liver disease as it has potential to be used as an early marker of detection for hepatocellular carcinoma HCC [116].

Moving forward, an important avenue for research lies in extending the exploration of cfRNA profiles pre- and post-bariatric surgery in individuals with or without metabolic complications. It seems that overall the majority of studies regarding cfRNA after bariatric surgery focus on the changes induced by the surgery *per se*. These changes may be associated with the weight loss and the amelioration of the metabolic phenotype. However, no studies exist on the predictive potential of pre-surgery cfRNA profiles of long-term (2, 5 and more years after the surgery) outcomes (weight loss, remission of diabetes, dyslipidemia etc). This type of research could be expanded also in patients undergoing treatment with the widely used obesity drugs, the GLP-1 analogs and ideally it could be predicted based on a liquid biopsy which patients would benefit more from a surgery or from a medical treatment or both. It could also help the physicians identify patients at high-risk of treatment/surgery failure and ensure a much stricter follow-up.

In conclusion, this review underscores the evolving landscape of non-invasive diagnostic methodologies, with a particular focus on liquid biopsies and specifically on cfRNA. The detection and monitoring of metabolic diseases with liquid biopsies, particularly those targeting cfRNA, hold significant promise. Examination of cfRNA signatures in various metabolic diseases, including diabetes, dyslipidemia, and fatty liver disease, contributes crucial insights into the molecular intricacies of these disorders. These cfRNA signatures may also be useful in the prediction of the outcome of a treatment or an intervention (bariatric surgery). Further, there needs to be greater understanding of the physiological levels of cfRNA and greater study into circadian rhythm, dietary influences, and responses to various physiological contexts. Studies exploring the circadian rhythmicity of circulating miRNAs and the diurnal variation of cfDNA and cfRNA illuminate the temporal dynamics that influence cfRNA profiles. Further investigations into cfRNA's role in lactation, fasting, and diverse physiological scenarios enhance its potential as a non-invasive health status indicator.

## Ethics approval and consent to participate

Not applicable.

## Consent for publication

Not applicable.

## Funding

This study was funded by the startup funds of I.G.S. from the 10.13039/100011594Penn State College of Medicine and by a Spark Grant (CRSK-3_220825) to D.V.C. by the Swiss National Science Foundation (SNSF). D.V.C. is supported by a University of Lausanne fellowship (Bourse de relève académique).

## CRediT authorship contribution statement

**Manvita Mareboina:** Writing – original draft, Visualization, Data curation. **Elen Deng:** Writing – original draft, Data curation. **Ioannis Mouratidis:** Writing – review & editing. **Nelson S. Yee:** Writing – review & editing. **Nelly Pitteloud:** Writing – review & editing. **Ilias Georgakopoulos-Soares:** Writing – review & editing, Writing – original draft, Validation, Supervision, Funding acquisition, Conceptualization. **Dionysios V. Chartoumpekis:** Writing – review & editing, Writing – original draft, Validation, Supervision, Funding acquisition, Data curation, Conceptualization.

## Declaration of competing interest

All authors declare that they have no conflicts of interest.

## Data Availability

No data was used for the research described in the article.

## List of abbreviations

cfDNA: cell-free DNA
cfRNA: cell-free RNA
CHC: chronic hepatitis C
DN: diabetic nephropathy
FH: familila hypercholesterolemia
GLP-1: glucagon-like peptide-1
HCC: hepatocellular carcinoma
MASLD: metabolic dysfunction-associated steatotic liver disease
NAFLD: Non-alcoholic fatty liver disease
RYGB: Roux-en-Y gastric bypass
STZ: streptozotocin
T1D: diabetes mellitus type 1
T2D: diabetes mellitus type 2
WHtR: waist to height ratio
